# Guided biofilm therapy versus conventional protocol—clinical outcomes in non-surgical periodontal therapy

**DOI:** 10.1186/s12903-024-04898-z

**Published:** 2024-09-18

**Authors:** Miriam Cyris, Julia Festerling, Maren Kahl, Claudia Springer, Christof E. Dörfer, Christian Graetz

**Affiliations:** Clinic of Conservative Dentistry and Periodontology, University of Kiel, Arnold-Heller-Straße 3, Kiel, 24105 Germany

**Keywords:** Biofilm, Dental polishing, Erythritol, Non-surgical therapy, Periodontitis, Scaling and root planing, Subgingival scaling

## Abstract

**Background:**

The aim of the randomized controlled clinical trial study was to evaluate the effectiveness in reducing pathologically increased pocket probing depths (PPD > 3 mm) using the Guided Biofilm Therapy (GBT) protocol (adapted to the clinical conditions in non-surgical periodontal therapy (NSPT): staining, air-polishing, ultrasonic scaler, air-polishing) compared to conventional instrumentation (staining, hand curettes/sonic scaler, polishing with rotary instruments) both by less experienced practitioners (dental students).

**Methods:**

All patients were treated according to a split-mouth design under supervision as diseased teeth of quadrants I/III and II/IV randomly assigned to GBT or conventional treatment. In addition to the treatment time, periodontal parameters such as PPD and bleeding on probing (BOP) before NSPT (T0) and after NSPT (T1: 5 ± 2 months after T0) were documented by two calibrated and blinded examiners (Ethics vote/ Trial-register: Kiel-D509-18/ DRKS00026041).

**Results:**

Data of 60 patients were analyzed (stage III/IV: *n* = 36/ *n* = 24; grade A/ B/ C: *n* = 1/ *n* = 31/ *n* = 28). At T1, a PPD reduction of all diseased tooth surfaces was observed in 57.0% of the GBT group and 58.7% of the control group (*p* = 0.067). The target endpoint (PPD ≤ 4 mm without BOP) was achieved in 11.5% for GBT (conventional treatment: 11.2%; *p* = 0.714). With the exception for number of sites with BOP, which was at T1 15.9% in the GBT group and 14.3% in the control group (*p* < 0.05) no significant differences between the outcomes of the study were found. At 30.3(28.3) min, the treatment time was significantly shorter in GBT than in the control group at 34.6(24.5) min (*p* < 0.001).

**Conclusions:**

With both protocols (GBT/ conventional instrumentation) comparably good clinical treatment results can be achieve in NSPT in stage III-IV periodontitis patients.

**Trial registration:**

The study was registered before the start of the study and can be found under the number DRKS00026041 in the German Clinical Trials Register. The registration date was 19/08/2021.

## Background

Scaling and root planing (SRP) remains to be the main treatment step in non-surgical periodontal therapy (NSPT) [1]. A wide variety of instruments are available for the removal of hard deposits as well as non-mineralized biofilm. However, effective, subgingival biofilm disruption faces certain limitations [2]. Classically, hand instruments such as curettes are used, but also sonic scalers which are operated by means of air pressure or ultrasonic scalers have always become established. For the selective removal of non-mineralized biofilm, conventional polishing with rotating instruments and polishing paste has long been available, though it is limited to supragingival areas. Therefore, the development of air-powder-devices with low abrasive powders was considered a paradigm shift in biofilm management, as this air polishing technology can be used both supra- and subgingivally [3]. Naturally, all mechanical instruments have their inherent advantages and disadvantages [4–7], and it is recommended to use a combination of different instruments to fully leverage their benefits or to compensate for their limitations [8].

A few years ago, the manufacturer EMS (Electro Medical Systems S.A., Nyon, Switzerland) introduced the Guided Biofilm Therapy (GBT), a new treatment protocol that systematically combines an air-abrasive device using erythritol powder and ultrasonic scalers with further interventions (e.g. use of plaque elevators) [9]. The GBT showed in the context of supportive periodontal therapy (SPT) or professional mechanical plaque removal (PMPR) treating a gingivitis comparable efficacy with the conventional approach [4, 9–13]. Conventional instrumentation using curettes, sonic scalers and rotary polishing is a frequently used method in NSPT [8, 14]. However, it is well known that SRP can have undesirable effects on hard and soft tissues [15], such as the loss of root cementum [16], microcracks in dentin [17], roughening of the tooth surface [18], hypersensitivity after treatment [19] and gingival recession [20]. Several studies have shown that these side effects of SRP are largely dependent on the user´s experience and training [21, 22], which is why proper training in these techniques is essential [23]. To reduce these complications in NSPT is a major challenge, especially as studies have detected that the removal of root cement is not necessarily required for successful treatment in NSPT [15].

Moreover, the use of subgingivally applicable powders in the air-polishing procedure offers the potential to influence the microbiome in the periodontal pockets though the substances used [24], although the oral microbiome of periodontitis patients differs from that of (peri-)implantitis patients. Studies have shown that glycine powder can significantly reduce the number of periodontal pathogenic bacteria, such as Porphyromonas gingivalis, compared to the application of a water–air mixture [25]. A systematic review also found that low-abrasive powders can also be beneficial in the treatment of peri-implantitis. In addition to the aforementioned aspect of protecting the dental tissue and soft tissue, the preservation of the implant surface can also be highlighted as an advantage [26].

Nevertheless, it should also be mentioned at this point that there are currently no national or international guidelines for the subgingival use of the powder-water jet technique on teeth [27]. With regard to its use on implants, the current evidence is even more inconsistent, as a distinction must be made between peri-implant mucositis or peri-implantitis, as well as between surgical or non-surgical treatment, and the combination with other instruments [28].

Thus, the GBT may be a useful adjunct in NSPT. Overall, there have been few studies comparing SRP with the GBT method during NSPT [7, 12, 29, 30]. To the best of our knowledge, no evidence currently exists regarding a learning curve for GBT in NSPT. Therefore, the aim of the present randomized controlled clinical trial (RCT) was to evaluate the efficacy of GBT in reducing pathologically increased pocket probing depths (PPD > 3 mm) compared to SRP in NSPT for patients with stage III and IV periodontitis by inexperienced practitioners under supervision. It was assumed (null hypothesis) that the GBT leads to significantly better clinical results than the conventional method for root surface cleaning in the NSPT.

## Methods

### Trial design

**Fig. 1:**
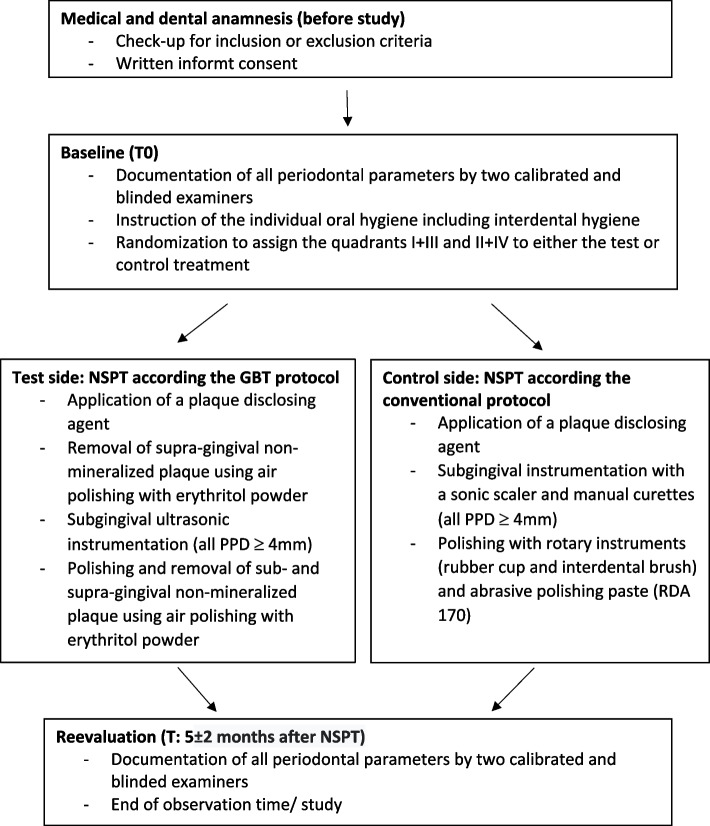
Treatment protocol of quadrants according the test (GBT) and control side (conventional). Legend: GBT: Guided Biofilm Therapy; NSPT: Non-surgical periodontal therapy; PPD: pocket probing depth; RDA: relative dentine abrasion

The RCT employed a split-mouth design to evaluate the clinical effectiveness of NSPT. The study compared two approaches: (1) GBT using low-abrasive erythritol powder in combination with a ultrasonic scaler (combined device Prophylaxis Master, E.M.S. Electro Medical Systems S.A., Nyon, Switzerland) versus (2) conventional SRP using hand instruments (Gracey curettes 5/6, 7/8, 11/12 and 13/14 (American Eagle Instruments, Missoula, MT, USA)) and/ or sonic scalers (Proxeo, W&H, Bürmoos, Austria) along with rotary polishing with rubber cups (Prophy-Cups, KerrHawe SA, Bioggio, Switzerland) and brushes (CRA 14 regular, Curaden, CURADEN GmbH, Stutensee, Germany) or an oscillating polishing device (EVA tip, Loser & Co GmbH, Leverkusen, Germany) for interdental areas. The conventional protocol is according internal treatment guidelines for NSPT in our department for several years [31], it´s also based on the current international recommendations for subgingival instrumentation to combine hand instruments with mechanical systems [27]. Two contralateral quadrants were randomized to be treated either with the GBT protocol (test side) or the conventional protocol (control side). This randomization followed the principle of simple unconstrained randomization (chance), performed using the Random Integer Set Generator tool on a website (https://www.random.org). The randomization also determined the treatment sequence for subsequent visits (Fig. 1). The randomization of the quadrants was carried out before the start of the study and kept in sealed envelopes. Upon the inclusion of a patient, a sealed envelope was opened, and the respective quadrants were treated according to the protocol specified by the randomization.

All interventions, within the framework of the first and second periodontal therapy steps [27], were performed by dental pre-graduate students (*n* = 100) of the third clinical semester, under supervision of experienced dentists (M.K., M.C., C.S., C.G.; all with more than five years of professional experience) over three semesters from April 2021 to December 2022. The participating students were systematically trained in the use of the different instruments, both theoretically and practically, on manikin heads and through mutual exercises [22, 32]. Two examiners (C.S., C.G.) were responsible for measuring the clinical data at T0 (baseline) and T1 (reevaluation of NSPT after 5 ± 2 months baseline) while the statistician (C.G.) was blinded to the treatment groups. No professional interventions occurred between the final subgingival instrumentation appointment and the reevaluation, which motivates patients to continue oral hygiene according to individual instructions during the latency period. Internal calibration for measuring periodontal parameters was performed by one dentist (C.S.) for both investigators before the clinical trial began. Test–retest exercises were performed with five subjects not involved in the trial to assess calibration between the investigators. A deviation of ± 1 mm for probing depths (PPD) and clinical attachment level (CAL) was considered acceptable. This measurement procedure has been previously published by our group (Sutor et al. 2024) [33].

### Participants

All participants signed a written informed consent form before inclusion. The study was approved by the Ethics Committee of the Christian-Albrechts-University (D 509/18) and registered in the German Clinical Trials Register (DRKS00026041) before the start of the study.

**Fig. 2:**
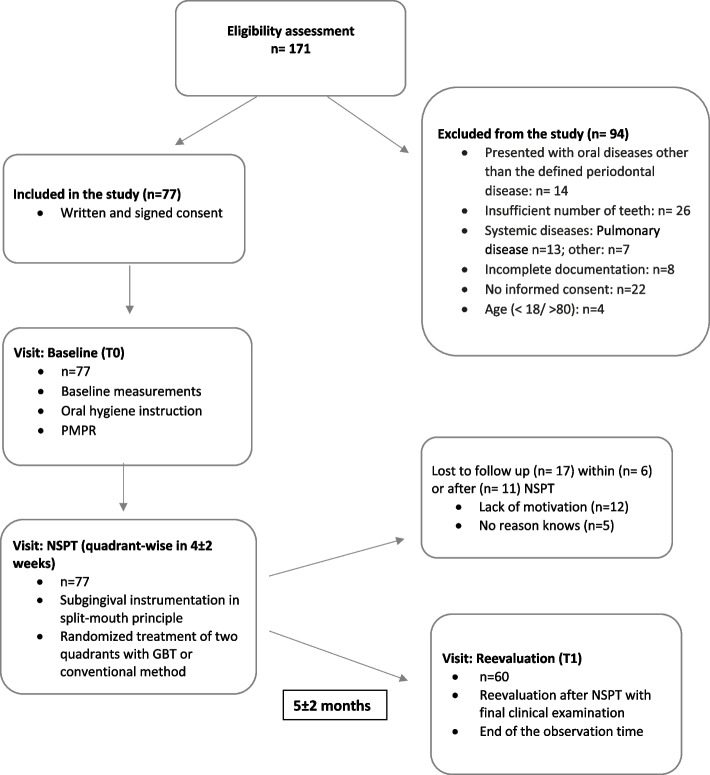
Flowchart of the patients’ recruitment and treatment protocol during the study. Legend: T0: baseline visit, T1: reevaluation 5 ± 2 months at the end of treatment phase of nonsurgical periodontitis therapy (NSPT), either quadrant treatment with ultrasonic scaler and air-polishing device (GBT) or quadrant treatment with conventional method such as curettes, air scaler and rotary polish (control)

Patients routinely participating in treatment for NSPT by the dental students were screened with regard to inclusion and exclusion criteria according the internal dental curricula (Fig. 2).

#### Inclusion criteria

- At least two teeth with PPD ≥ 4 mm in at least four quadrants
- Generalized periodontitis with stage I or higher, patient age 18–80 years) and

#### Exclusion criteria

- Systemic diseases requiring antibiotic prophylaxis due to systemic bacteremia during dental treatments (e.g. chemotherapy and/or radiotherapy, endocarditis)
- Patient age < 18 or > 80 years
- Patient's lack of consent to student treatment
- Respiratory diseases (bronchial asthma, chronic bronchitis, chronic obstructive pulmonary disease (COPD))
- Acute infections
- Pregnant and breastfeeding women
- Allergies to erythritol or chlorhexidine

### Intervention protocols for nonsurgical periodontal therapy

According the recommendations of a systematic periodontal treatment [27], at baseline, all patients received center-standard instructions on individual oral hygiene and PMPR. Specifically, patients were instructed on the proper use of a toothbrush (case-by-case decision: manual toothbrush or oscillating-rotating toothbrush) twice daily, and in the use of interdental brushes with adapted sizes for each interdental space. NSPT was performed at individualized intervals in quadrants over a period of 4 to 6 weeks (depending on center and patient scheduling priorities). Subgingival instrumentation was usually performed under local anesthesia (SOPIRA® Citocartin® (Active ingredient: Articaine), Kulzer GmbH, Hanau, Germany), unless the patient explicitly refused local anesthesia; these cases were documented for the statistical analysis of the patients' perception of pain. Other treatments directly affecting the treatment outcome, such as adjunctive use of antibiotics or antibacterial agents, were not performed by the practitioner during/after NSPT or by the patients at home (Figs. 1 and 2).

Quadrants randomized for GBT protocol received the following treatment:

- Plaque coloring all teeth to determine plaque levels and to guide or optimize biofilm removal.
- Removal of supra-gingival non-mineralized plaque using air polishing with erythritol powder.
- Tooth surfaces requiring treatment (PPD ≥ 4 mm) were instrumented with an ultrasonic scaler with a slime-line tip (PERIO SLIM PIEZON PS, EMS, Nyon, Switzerland) throughout the randomized quadrant, including the removal of supragingival calculus.
- Removal of non-mineralized biofilm in the supra- and subgingival areas were performed with erythritol powder (PLUS® powder, EMS, Nyon, Switzerland). It should be noted, that independent the depth of PPD no nozzle handpiece was utilized to prevent emphysemas in NSPT according the manufactory recommendations.

The amount of powder is dosed independently by using the system AIRFLOW® Prophylaxis Master (EMS, Nyon, Switzerland)—individualization by the user is not possible. The amount of water was kept constant at level 10 and the intensity was adjusted according to the patient's individual clinical findings and the manufacturer's recommendations.

Quadrants randomized for conventional protocol received the following treatment:

- Plaque coloring all teeth to determine plaque levels and to guide or optimize biofilm removal.
- Tooth surfaces requiring treatment (PPD ≥ 4 mm) were instrumented with sonic scaler (Proxeo, W&H, Bürmoos, Austria) on level 2 according manufactory recommendations with a slime-line tip (1AP, W&H, Bürmoos, Austria) in combination with manual instrumentation (Gracey curettes, American Eagle Instruments, Missoula, MT, USA).
- Removal of supragingival non-mineralized biofilm was performed with rubber cups and abrasive polishing paste (ProphyCare Prophy-Paste CCS Polishing Paste medium, relative dentin abrasion (RDA) 170) and various devices for interdental areas as mentioned before.

### Outcomes

The following parameters were measured at T0 and T1; PPD, gingival recession (GR), and bleeding on probing (BOP). The CAL was calculated with the results of PPD and GR. All parameters were recorded at 6 points per tooth using the UNC-PCP 15 probe (Hu-Friedy®, Chicago, IL, USA). After coloring the plaque with a disclosing agent (Mira-2-Ton®, Hager & Werken, Duisburg, Germany) a plaque index according to O’Leary (PCR) was documented. BOP and PCR were recorded as dichotomous index (presence/absence) and afterwards calculated in a percent value. Beside these parameters, treatment time per quadrant and patients' perception of pain during and after the interventions, categorized using a visual analog scale (0 (no pain) to 10 (most severe pain imaginable)), were documented. The treatment time was defined as the time spent actively working with the various instruments until the pre-graduate student or the supervisor were satisfied.

The primary endpoint was the change in PPD at tooth surface level from baseline (T0) to T1 after NSPT, i.e., the percentage distribution of which tooth surfaces became closed pockets (PPD ≤ 4 mm without BOP). Secondary outcomes were the changes of GR, CAL, BOP, treatment time and patients' perception of pain.

### Statistical analysis and sample size

Statistical analysis was performed using SPSS Statistics 24 software (IBM, Chicago, IL, USA) for multilevel statistical analysis at tooth surface level, tooth level, and patient level.

Prior to the study a power calculation was performed. In view of the lack of comparative studies with GBT in NSPT, the calculation of the sample size based on the results published by Geisinger et al. (2007) with two treatment groups (with versus without a perioscopy in NSPT) and showed that *n* = 50 teeth per group would be sufficient to detect a difference of less than five percent (power of 80%, alpha 0.05, Beta 0.2) for the primary outcome (proportion of PPD ≤ 4 mm without BOP). The expected mean of proportion of PPD ≤ 4 mm was 80% versus 90% respectively (expected mean difference (standard deviation): 10 (12)).

Therefore, 15 patients with at least n ≥ 16 teeth were considered sufficient for the present split-mouth study. To account for failures, especially recording possible imbalanced data according the split mouth design, the fourfold number of patients was chosen. Therefore, an overpower was deliberately accepted with a large sample size and maybe waste of resources, nevertheless, as the study ran over several semesters, patients were included consecutively and different students repeatedly performed both treatment methods on the patients, so that in the end 60 patients, took part in the study.

Normality of data was tested using Kolmogorov–Smirnov and Shapiro–Wilk tests. Data at the tooth level were not normally distributed (Kolmogorov–Smirnov test/Shapiro–Wilk test: *p* < 0.001/*p* < 0.001). Therefore, within-group changes were assessed using the nonparametric Wilcoxon-Test and between-group differences were assessed using the Mann–Whitney-U-Test. All statistical tests were two-sided, and a value of *p* < 0.05 was considered significant.

## Results

### Recruitment

One hundred seventy-one patients were screened for the study, and a total of 77 patients were selected. During the study, 17 patients were excluded (dropout) because 6 patients did not attend all appointments for NSPT or 11 patients did not attend the T1 appointment (Fig. 2). Thus, 60 patients could be included in the analysis. The mean (SD) observation between T0 and T1 was 4.47 (1.6) months.

### Demographic and baseline data

Thirty-four female and 26 male patients participated in the study. The mean (SD) age was 46.34 (11.67) years. 16 participants were smokers. 3 patients had diagnosed diabetes mellitus. Staging and grading were distributed as follows: Stage III (36), Stage IV (24); Grade A (1), Grade B (31), and Grade C (28), with 10 patients having a localized extent, 49 having a generalized extent, and one patient showing a molar incisor pattern (Table 1). For subgingival instrumentation intraoral anesthesia were utilized as block anesthesia in 40.8% and infiltration anesthesia in 35.1%. 24.1% of all treated teeth were not local anaesthetized.

**Table 1:** Patient’s characteristics at baseline

Parameter	Percent (%)
**Sex**	
Female	56.7
Male	43.3
**Smoking dichotomy**	
No smoking	73.3
Smoking	26.7
**Diabetes mellitus**	
No diabetes	95.0
Diabetes	5.0
**Staging**	
Stage 3	60.0
Stage 4	40.0
**Grading**	
Grade A	1.7
Grade B	51.7
Grade C	46.7
**Extent**	
Localized	16.7
Generalized	81.7
Molar incisive pattern	1.7

Table 2 showed an overview about baseline parameters at T0, subdivided for GBT versus control group and did not differ significantly from each other.

**Table 2:** Evaluation from descriptive and statistical analysis at tooth level (T0 and T1) and parameters at baseline (T0) and after treatment (T1) (average)

Parameter	Control group	GBT group	Difference between treatment groups
**Tooth type (%)**			***p*-value**
Front	48.1	48.6	0.833
Premolar	29.0	28.9	
Molar	22.9	22.5	
**Tooth mobility T0 (%)**			
No movement	86.4	85.2	0.469
Degree I	11.3	10.9	
Degree II	2.1	3.5	
Degree III	0.3	0.5	
**Tooth mobility T1 (%)**			
No movement	91.8	90.4	0.35
Degree I	5.7	6.1	
Degree II	1.9	3.2	
Degree III	0.6	0.3	
	**mean (SD)**	**mean (SD)**	
PDD at T0 [mm]	3.8 (1.7)	3.8 (1.6)	0.289
GR at T0 [mm]	1.0 (1.3)	1.0 (1.3)	0.22
CAL at T0 [mm]	4.7 (2.1)	4.6 (2.0)	0.632
Visual Analog Scale for pain during treatment	2.5 (2.4)	2.4 (2.1)	> 0.05
PDD at T1 [mm]	2.9 (1.3)	2.9 (1.2)	0.23
GR at T1 [mm]	1.2 (1.5)	1.2 (1.4)	0.825
CAL at T1 [mm]	4.0 (2.0)	4.1 (2.9)	0.284
PDD-difference T1-T0 [mm]	-0.9 (1.4)	-0.9 (1.3)	0.029*
GR-difference T1-T0 [mm]	0.2 (1.1)	0.3 (1.0)	0.299
CAL-difference T1-T0 [mm]	-0.6 (1.8)	-0.6 (1.7)	0.148
treatment time per group for quadrants [min]	34.6 (24.5)	30.3 (28.3)	< 0.001*
Visual Analog Scale for pain post-op	0.1 (0.4)	0.1 (0.3)	> 0.05

T0: baseline visit, T1: reevaluation after the treatment phase of nonsurgical periodontitis therapy (NSPT), *GBT* Guided Biofilm Therapy, *PD* pocket probing depth, *GR* gingival recession, *CAL* clinical attachment loss; all Mann–Whitney-U-Test

^*^Indicates significant values

### Results on patients’ level

All clinical parameters PPD, GR and CAL at T0 and T1 as well as the difference of GR and CAL between T0 and T1, showed no significant differences between treatment groups (*p* > 0.05). On the other hand, T0 to T1 changing of PPD (*p* = 0.029) as well as the treatment time per quadrant (*p* < 0.001), differed statistically significant between both groups. The mean treatment time required in GBT and control treatments differed significantly of 4.3 min per quadrant. On average, 34.6 min per quadrant were required for subgingival cleaning in the control group and 30.3 min in GBT (Table 2). On patients level no significant differences between GBT and control could be detected for pain sensation during (*p* =  > 0.5) and after treatment (*p* =  > 0.5).

The pain perception of the patients was assessed by the patients themselves using the VAS-analog scale, the mean value during treatment in the control group was 2.5 (2.4) in the GBT group on average 2.4 (2.1), in both groups the pain perception under local anesthesia was in the lower third of the scale and there was no significant difference between the two groups (*p* > 0.05). The perception of pain after treatment was reported in both groups with an average of 0.1 (Table 2).

### Results on tooth level

In Table 3 were all results on tooth level summarized. In terms of PPD at T0, 53.5% of the tooth sites in the control group were ≤ 3 mm, 30.8% were 4–5 mm, 15.7% were ≥ 6 mm and in the GBT group were 54.7% with ≤ 3 mm, 30.1% with 4–5 mm, and 15.2% with ≥ 6 mm, respectively. At T1, we found for both treatment protocols a significant increase of the sites with PPD ≤ 3 mm (*p* < 0.001), whereas without significant difference (*p* > 0.05) between both groups (Table 3). A significant decreasing percentage (*p* < 0.001) of sites with PPD of 4–5 mm and PPD ≥ 6 (*p* < 0.001) were found. Beside these significant changes after NSPT (T0-T1) in both groups, no statistically significant differences were detected between both (*p* > 0.05).

**Table 3:** Statistical analysis of the parameter bleeding on probing (BOP) and pocket probing depth (PPD) subdivided in test (GBT) and control-group (conventional treatment)

	**Control group (in percent)**	**GBT group (in percent)**	**Difference between treatment groups**
**Pocket probing depth T0**			***p*-value**
≤ 3 mm/4-5 mm/ ≥ 6 mm	53.5/30.8/15.7	54.7/30.1/15.2	0.286
**Pocket probing depth T1**			
≤ 3 mm/4-5 mm/ ≥ 6 mm	80.7/14.3/5.0	81.0/14.1/4.9	0.732
**BOP T0**			
No bleeding/ bleeding	57.1/34.8	53.6/34.9	0.155
**BOP T1**			
No bleeding/bleeding	81.1/14.3	78.9/15.9	0.037*
**PPD ≤ 4 mm and BOP at T1**			
Yes/ no	88.8/11.2	88.5/ 11.5	0.714
**BOP change between T0-T1**			
Decrease/stable/increase	29.5/63.0/7.5	29.2/62.9/7.9	0.621
**PPD change between T0-T1**			
Decreased/unchanged/increased	58.7/33.3/7.9	57.0/33.9/9.1	0.067

T0: baseline visit, T1: reevaluation after the treatment phase of nonsurgical periodontitis therapy (NSPT), *GBT* Guided Biofilm Therapy, *BOP* bleeding on probing, *PPD* pocket probing depth; all Mann–Whitney-U-Test, ^*^Indicates significant values

With regard to BOP, at T0, BOP was present on 34.8% of all tooth sites in the control group and 34.9% in the GBT group (*p* = 0.155). At T1 after NSPT, BOP was still present on 14.3% of all tooth sites in the control group versus 15.9% in GBT (*p* = 0.037). In contrast, when analyzing the BOP change during T0 to T1 or the number of PPD of 4 mm and BOP at T1, no significant differences between both groups could be determined (BOP: *p* = 0.621, PPD of 4 mm and BOP: *p* = 0.714; Table 3). Therefore, except for the percentage value of BOP at T1, no statistical differences in clinical parameters between both treatment groups could be measured.

In addition, no significant differences between both treatment groups were found for the distribution of tooth type and the tooth mobility at T0 and T1 (Table 2).

## Discussion

This clinical study was designed to determine possible differences in clinical outcome when conventional subgingival instrumentation with hand/ powered instruments and rotating rubber cups were used compared with the GBT approach during NSPT of patients with stage III-IV periodontitis. Both, GBT and conventional treatment protocol, resulted in significant improvement of all periodontal parameters surveyed.

At the time of re-evaluation, approximate five months after NSPT, 95.1% of the GBT trial sites and 95.0% of the control group trial sites achieved a PPD ≤ 5 mm (PPD ≤ 4 mm GBT/ control at T1: 91.1%/ 90.9%), in consequence meaning that no further surgical therapy is indicated at these sites [27]. Overall, these results compare very favorably with other achieved goals after nonsurgical periodontal therapy described in the literature. Wennström et al., [34] achieved after hand instrumentation in 66% and in 58% after application of ultrasonic scaler in the sense of full-mouth scaling, pocket closure (PPD ≤ 4 mm) after a period of three months. After six months, the value increased to 77% and 74%, respectively. In the present study, a greater decrease in PPD was already achieved after a mean of five months with both protocols. Additionally, in comparison with a recent systematic review, the achieved percentage pocket closure was higher than the mean value of 74% for subgingival instrumentation with different instruments such as manual, ultrasonic or sonic scaler [8]. However, the work of Suvan et al. [8] also concluded that a combination of different instruments achieves the best and most predictable results in subgingival instrumentation, which was also applied in our study.

Our results emphasize that combining and using different instruments in NSPT requires more complex education and training but allows the practitioner to select the optimal instrument for different indications or treatment cases. For example, while air-powder devices lack tactile sensation, they minimize trauma to surrounding soft tissue [35]. Curettes, although potentially more invasive, allow for precise removal of granulation tissue. It is also assumed that not all practitioners would commit to one instrument combination or one propagated system. Economic factors (regulations in national healthcare systems, guidelines, costs for several instruments, etc.) or availability (pandemic-related restrictions, etc.) may influence even experienced users to favor individual instruments. Nevertheless, our study still shows slightly better results than those reported by Suvan et al. [8]. Possible reasons could be the heterogeneity of the included studies, the variety of instruments, protocols and combinations considered. However, it should be noted—our both protocols did not differ with regard to the percentage of closed pockets after NSPT at T1.

Otherwise, the BOP at T1 was with 15.9% for GBT significantly higher than 14.3% for the control group (*p* = 0.037). Whereas this difference of approximately 1.6% was certainly not clinically relevant, nevertheless, it represented a statistically significant difference. According to the current classification of periodontal and gingival disease of 2018 [36], stable periodontal conditions are characterized by a BOP that is < 10%, and no probing depth of 4 mm or more that bleeds on probing [37]. In the present study, the overall BOP at T1 is not that far from the BOP < 10% target, at 14.3% and 15.9%, respectively. This result, showing a greater BOP reduction in the control group, is consistent with findings from another recent study that analyzed the effect of both methods in SPT [38]. In that study, the BOP decreased from 14.7% in the conventional treatment to 7.9% (GBT from 12.2% to 9%), with significant difference between both treatment groups [30]. This suggests that, in addition to the effective systematic treatment of the root surface, the repeated instruction and motivation of oral hygiene seems to have had a positive effect and also represents a central component in the treatment guidelines [27]. This effect is also supported by the positive development of the PCR from 59.2 (23.5) % at T0 to 43.1 (22.4) % at T1.

Nevertheless, the professional interventions in NSPT seems to have an important influence, as the BOP values are also better as mentioned before. The systematic review of Suvan et al. [8] determined a BOP reduction of 62% at six and eight months, respectively, which is already close to the reduction in our work of 41.09% in the control group and of 45.5% in the GBT group, which was achieved in approximately five months. However, this percentage ratio also indicates that despite the significant differences in BOP at T1, the percentage ratio of BOP reduction within the GBT group, GBT was even higher than within the control group.

In comparison with other study results of the use of air-polishing method during periodontal treatment phases [12, 30, 39–41], all of them showed similar results, with all of them showing an improvement in clinical parameters, but overall, no significant clinical advantage of the air-polishing method was observed. However, a very specific comparison is difficult because of the wide variability of materials and protocols used in these studies. Park et al. [40] and Jentsch et al. [30] used the same erythritol powder and technique as in the present study, however, we used it not only on the tooth surfaces with increased probing depths but on all teeth of the instrumented quadrant (supra- and subgingival), which is in accordance with the manufacturer's recommendations. Also, Tsang et al. [41] and Caygur et al. [39] used a glycine-based powder on specific sites with increased probing depths after combined instrumentation with ultrasonic scaler and curettes. Thus, all these studies investigated the air-polishing method only as an adjunct therapy to traditional subgingival instrumentation, but not the effect of the GBT protocol compared to conventional protocols with combined instrumentation. Flemmig et al. [29] measured a significant reduction in subgingival biofilm and a positive shift in the microbiota after the use of air-polishing (glycine-based) in periodontal pockets and on the mucosa for the entire mouth compared with hand instrumentation. It seems quite possible that the use of mechanical instruments in both groups in our study removed more hard and soft deposits than would have been possible with manual instrumentation alone [8]. Additionally, it has been proven that machine instruments allow better access to anatomical features such as furcation or root retractions [42–44] and the rinsing function might have additionally flushed effects of the bacterial residues and endotoxins [45].

Alongside treatment results, it is worth noting that the use of the GBT protocol compared to the conventional approach resulted in a significantly shorter mean time of 4.3 min in our study setting (this corresponds to a treatment time reduction of 12.43%). This result is in accordance with other GBT studies with 9.5% reduction in treatment time for patients with gingivitis [46] or Vouros et al. [11], which showed an average time saving of 5.1 min in SPT. Conversely, the differences described represent a time saving. Although this small difference will not shorten the total treatment time in the NSPT for the patient or dentist, the time gained can possibly be used for additional explanations, e.g. about oral hygiene or risk factors for periodontitis, as described in the current international treatment recommendation [27]. No side effects of air-polishing were observed (e.g., swelling, or emphysema) and the pain perception of the patients under local anesthesia in both treatment groups was, as expected, in the lower third of the VAS scale during treatment (GBT/ control: 2.5 (2.4)/ 2.4 (2.1); *p* > 0.05) and nearly zero after NSPT (GBT/ control: 0.1 (0.3)/ 0.1 (0.4); *p* > 0.05). Nevertheless, it should be noted here that emphysema can occur when using air-polishing, particularly treating implants, as has already been described several times in the literature [47, 48]. In vivo studies also suggest that air-polishing, even with low-abrasive powders, can lead to damage to the gingiva depending on the working angle [49], in addition to a loss of substance at restoration margins [50] with unpredictable long-term effects e.g. for pain/ hypersensitivities or root caries.

### Limitations

Despite the strengthen of the current RCT, several limitations must be acknowledged. (1) Although the split-mouth design with treatment assigned by quadrant eliminates interindividual variability (smoking status, oral hygiene) in clinical outcomes, in carries a a higher risk of a carryover effect [51]. To mitigate this, we decided to overpower our study by including nearly the fourth more patients than necessary. Furthermore, the current results cannot be generalized because (2) the observation period was limited to 5 ± 2 months. It is assumed that a longer observation time period would result in greater clinical attachment gains or at least fewer instances of recurrence requiring surgical periodontal therapy [52]. Otherwise, for patients with periodontitis stage I to II, which we failed to recruit for the current study, this shorter observation time after NSPT will be suitable, as no surgical intervention or complication are to be expected [27]. (3) The exclusion of patients with COPD will have reduced the number of participants even COPD and periodontitis share similar confounders e.g. smoking [53] and both are associated with each other [54]. However, according treatment guidelines for the dental curricula of our department, COPD, asthma bronchial etc. are contraindicated to use airflow devices to avoid wheezing and bronchospasm. Similarly, excluded patients with systemic diseases requiring antibiotic prophylaxis or with allergies to ingredients of the test products (e.g. erythritol, chlorhexidine) must also interpreted as a selection bias. Additionally, we did not perform a subgroup analysis of the 14 ‘smokers’ due to an imbalanced statistical distribution. Generally, smoking negatively impacts periodontal therapy, and we speculated that the powder-water jet might be less effective at reaching the tooth surface in smokers, as their gingiva is often thicker or coarser. This question should be focused in future investigations selectively. Additionally, the chlorhexidine contained in the air flow powder for GBT is used as a preservative (0.3%). At a concentration of 0.3% of the powder weight, chlorhexidine does not act as an adjuvant therapeutic agent and the mixture with water leads to additional dilution. The influence of chlorhexidine can therefore be neglected in principle. However, it is possible that its delayed release from the mucosa could have additionally influenced healing in the control quadrants [46, 55–57]. However, only a handpiece with supragingival nozzle was used in the study, so a deeper introduction of the active ingredient chlorhexidine into the treated periodontal pocket can be ruled out. (4) The treatment time and pain measurement using VAS also represent limitations of this study. Different numbers of teeth in the treated quadrants, as well as the use of local anesthesia, may have led to the NSPT being performed for different lengths of time in patients by similar pain perception, regardless of the protocol used. The pain measurement with VAS is a subjective measurement method and thus a study-related limitation and was recorded in the patients despite regular local anesthesia, which may have led to a distortion of the pain tolerance; in general, only a very low pain level could be determined during the NSPT. A last critical point to be mentioned, (5) according the recommendations of the first periodontal treatment step [27], control of factors such as patients compliance and self-performed oral hygiene was performed by the pre-graduate students. However, poor plaque scores or small improvements will affect the treatment outcomes individually, why a randomization and split-mouth design were chosen to minimize this effect. Overall, future clinical studies with a parallel design and even larger cohort will help to clarify these issues, and also allow for the analysis of other aspects, such as changes in the microbiome.

## Conclusion

GBT, when compared to the conventional protocol, good clinical treatment results according to the percentage of pocket closure in NSPT can be achieved on patients with stage III-IV periodontitis, even by systematically trained pre-graduated students under supervision. Conventional protocol showed slightly more favorable results regarding BOP after NSPT but required significantly more time for treatment, with comparable subjective pain perception reported by patients.

## Data Availability

The datasets used and analyzed during the current study are not publicly available due to [national data protection law] but are available from the corresponding author on reasonable request.

## Abbreviations

BOP: Bleeding on probing
CAL: Clinical attachment level
COPD: Chronic obstructive pulmonary disease
GBT: Guided Biofilm Therapy
GR: Gingival recession
NSPT: Non-surgical periodontal therapy
PCR: Plaque index according to O’Leary
PPD: Pocket probing depths
PMPR: Professional mechanical plaque removal
RCT: Randomized controlled clinical trial
RDA: Relative dentin abrasion
SRP: Scaling and root planing
SPT: Supportive periodontal therapy
